# Visceral to subcutaneous fat ratio as an indicator of a ≥30% eGFR decline in chronic kidney disease

**DOI:** 10.1371/journal.pone.0241626

**Published:** 2020-11-16

**Authors:** Hiroshi Kataoka, Toshio Mochizuki, Kazuhiro Iwadoh, Yusuke Ushio, Keiko Kawachi, Saki Watanabe, Kentaro Watanabe, Taro Akihisa, Shiho Makabe, Shun Manabe, Masayo Sato, Naomi Iwasa, Rie Yoshida, Yukako Sawara, Norio Hanafusa, Ken Tsuchiya, Kosaku Nitta

**Affiliations:** 1 Department of Nephrology, Tokyo Women’s Medical University, Tokyo, Japan; 2 Clinical Research Division for Polycystic Kidney Disease, Department of Nephrology, Tokyo Women’s Medical University, Tokyo, Japan; 3 Department of Blood Purification, Tokyo Women’s Medical University, Tokyo, Japan; International University of Health and Welfare, School of Medicine, JAPAN

## Abstract

Whether the visceral-to-subcutaneous fat ratio (V/S ratio) is associated with renal prognosis in patients with chronic kidney disease (CKD) remains unclear. Furthermore, little is known about the effect of sex and the absolute amount of visceral fat accumulation such as visceral fat area (VFA) ≥100 cm^2^ on the V/S ratio in relation to renal prognosis. In this study, 200 patients with CKD were evaluated for renal prognosis. Survival analyses and logistic regression analyses were conducted, generating time-series pseudo-*R*^2^ values. The mean and percent change of the pseudo-*R*^2^ values from the 6^th^ year to the 10^th^ year (6Y–10Y Mean and 6Y–10Y Change, respectively) were calculated for determining the cut-off points for the medium-term renal prognosis. Multivariate Cox regression analysis revealed that the V/S ratio was significantly associated with renal outcomes and that the VFA category (VFA ≥ 100 cm^2^) had significant interactions with the V/S ratio regarding renal prognosis. The hazard ratio (HR) of the V/S ratio was higher in the sub-cohort of VFA **<** 100 cm^2^ than in the sub-cohort of VFA ≥ 100 cm^2^ (HR: 6.42 vs. 1.00). Regarding sex differences, a strong association was noted between the V/S ratio and renal prognosis in women but not in men (HR: 2.40 vs. 1.10). On the other hand, 6Y–10Y Mean of the pseudo-*R*^2^ values indicated differences in the cut-off points of the V/S ratio between men and women (V/S ratio: 0.75 vs. 0.5). Our findings indicate that it may be clinically meaningful to consider the differences in sex and the amount of VFA ≥100 cm^2^ for the V/S ratio in relation to renal outcomes in patients with CKD. The 6Y–10Y Mean of the pseudo-*R*^2^ values contributed to determining the cut-off points of the V/S ratio according to the sex difference.

## Introduction

Visceral fat accumulation is thought to be the most important central pathological condition in metabolic syndrome (MetS) [1, 2] and is strongly associated with metabolic disorders and cardiovascular disease (CVD) [3]. In contrast, subcutaneous fat is potentially beneficial against metabolic disorders [4]. The visceral-to-subcutaneous fat ratio (V/S ratio) is a combined index of visceral and subcutaneous fat and has strong associations with cardiometabolic risks [5, 6]. For example, in the Framingham Heart Study, the V/S ratio was found to significantly correlate with cardiometabolic risk factors such as blood pressure, dyslipidemia, and insulin resistance, beyond its associations with the body mass index and visceral adipose tissue [5]. Additionally, the V/S ratio has been associated with CVD events [7, 8].

Furthermore, obesity-related or adiposity-related glomerulopathy has been attracting attention. Increased production of proinflammatory adipokines is involved in the pathogenesis of CKD, causing oxidative stress, inflammation, endothelial dysfunction, hyperfiltration, activation of the sympathetic and renin–angiotensin systems in the kidneys, and insulin resistance [9–14]. Histological glomerular hypertrophy [15–22], which represents glomerular inflammation and glomerular hyperfiltration, is recognized as an early marker of obesity-related or metabolic kidney damage [22–26]. However, to our knowledge, there is no report clearly demonstrating an association between the V/S ratio and renal prognosis in patients with CKD. In June 2020, we conducted a literature search of the PubMed database using the keywords “renal prognosis” and “visceral to subcutaneous fat ratio”; only 13 relevant articles were identified, and none of the studies found a statistically significant association between the V/S ratio and CKD progression. Although the reasons for this are unknown, we consider it meaningful to consider the influence of sex or the amount of abdominal fat accumulation. Indeed, the V/S ratio has been reported as greater in men than women (0.84 vs. 0.39, respectively) [5], greater in obese individuals than in nonobese individuals (0.55 vs. 0.50, respectively), and greater in patients with MetS than in patients without MetS (0.69 vs. 0.48, respectively) [8]. Moreover, the effect of the absolute amount of visceral fat accumulation on the V/S ratio should also be considered.

Considering the aforementioned differences in the V/S ratio, research on renal prognosis evaluating sex differences and differences according to abdominal fat accumulation is desired. Furthermore, considering that cut-off values are needed to guide clinical decision-making [27], research evaluating the appropriate cut-off values for various sub-cohorts is desired. We believe that an analysis of the time-series changes in pseudo-*R*^2^ values [19, 21] is suitable in the context of these considerations. Variables that maintain a high pseudo-*R*^2^ value during follow-up or show an increasing pattern in pseudo-*R*^2^ values over time are considered useful as medium-term and long-term prognostic factors [19]. In the present study, we hypothesized that the renal pathophysiology of the V/S ratio differs according to sex or abdominal fat accumulation. Therefore, we examined sex differences and differences according to visceral fat accumulation in the medium-term time-series changes in pseudo-*R*^2^ values for the renal prognosis using several V/S ratio cut-off values in patients with CKD.

## Subjects and methods

### Ethics statement

The patients’ human rights and methods for protecting their personal information were considered in detail. All the relevant and responsible staff adhered to the principles of the Helsinki Declaration (amended October 2013) and the Ethical Guidelines for Clinical Studies (revised February 28, 2017; referred to hereafter as the Clinical Studies Ethical Guidelines) in the execution of this study. This cohort study was approved by the Medical Ethics Committee of Tokyo Women’s Medical University (#4599). All participants gave their informed consent at the time of entry.

### Patient selection

We reviewed the data of 2012 outpatients with CKD who visited the Kidney Center at Tokyo Women’s Medical University Hospital in Japan between August 2006 and August 2007. Among these, 201 patients without nephrotic syndrome underwent abdominal computed tomography (CT). After excluding one patient with nephrotic syndrome, the remaining 200 were enrolled in the present study. CKD was diagnosed according to previously described criteria [28].

### Covariate assessments

During a regular outpatient clinic visit, anthropometric and physical examinations were conducted, including assessments of blood pressure components, body height, body weight, visceral fat area (VFA), and subcutaneous fat area (SFA). The VFA and SFA were measured on images obtained by a multidetector-row CT examination using a GE LightSpeed scanner (General Electronics Healthcare, Milwaukee, WI, USA). An index image was obtained before scanning, and the umbilicus to the L4–5 level was identified as previously described [29, 30]. Horizontal images were obtained at 400 mA and 120 kVp, with a scan time of 1.0 s. The range of CT values covered the optimal CT numbers for adipose tissue (i.e., from −150 to −40.14). Data were stored and analyzed on a GE advantage workstation Ver. 4.0 (General Electronics Healthcare). For the V/S ratio, we evaluated eight cut-off values of interest (0.25, 0.3, 0.5, 0.55, 0.7, 0.75, 1.0, and 1.25). Blood pressure was measured in triplicate using a mercury sphygmomanometer; the average value was used in analyses. All biochemical analyses were performed on samples obtained after an overnight fast. Serum creatinine levels were measured enzymatically. The estimated glomerular filtration rate (eGFR) for Japanese patients was calculated using a previously described formula [31]. Concomitant drug use (antihypertensive drugs, diuretics, and drugs for the treatment of hyperuricemia, dyslipidemia, and diabetes mellitus) and comorbidities at entry were also assessed. Hypertension was defined as systolic blood pressure ≥ 140 mmHg, diastolic blood pressure ≥90 mmHg, or taking antihypertensive medication. Hyperuricemia was defined as serum uric acid level ≥7.0 mg/dL or taking antihyperuricemic medication. Hyperglycemia was defined as a blood glucose level of ≥110 mg/dL. Hypertriglyceridemia was defined as serum triglyceride level ≥150 mg/dL or taking oral lipid-lowering medication. Hypercholesterolemia was defined as serum total cholesterol level ≥220 mg/dL, serum low-density lipoprotein cholesterol level ≥140 mg/dL, or taking oral lipid-lowering medication. Low high-density lipoprotein (HDL) cholesterol was defined as a serum HDL cholesterol level ≤40 mg/dL. Diabetic kidney disease, chronic glomerulonephritis, and nephrosclerosis were diagnosed either from biopsies or clinically by the doctor in charge. The participants were followed up until December 31, 2019.

### Study endpoint

The study’s endpoint was kidney disease progression, which was defined as a ≥30% decline in the eGFR from baseline (≥30% eGFR decline) or the development of end-stage renal disease (ESRD) requiring renal replace therapy [32].

### Statistical analysis

Continuous variables are expressed as means and standard deviations (SDs) or as medians (quartile 1, quartile 3). Categorical variables are expressed as percentages, unless otherwise stated. Group differences were evaluated using the unpaired Student's t-test, Mann–Whitney U test, chi-square test, or Fisher’s exact test, as appropriate. Logistic regression analyses were used to assess the goodness of fit of renal prognostic models. The goodness of fit was assessed using McFadden’s pseudo-R-squared (pseudo-R^2^) [33], which is used for time-series evaluations. The annual percentage change in pseudo-*R*^2^ values (%/year) was calculated as follows: % pseudo-*R*^2^ change = pseudo-*R*^2^ slope/baseline pseudo-*R*^2^ ×100. The pseudo-*R*^2^ slope was calculated by the least-squares method. Renal prognostic factors were also evaluated in Cox regression analyses, and the Kaplan–Meier method was used for survival analyses. We followed standard methods to estimate the sample sizes for the multivariate logistic and Cox regression analyses; at least five outcomes were needed for each included independent variable [34]. Interactions between sex (male, female) or VFA category (≥100 cm^2^, <100 cm^2^) and each variable of interest were considered by adding sex or VFA category and the corresponding interaction term to the multivariate Cox proportional hazards model. *P*-values <0.05 were considered statistically significant. All statistical analyses were performed using JMP Pro software, Windows v15.0.0 (SAS Institute, Cary, NC, USA).

## Results

### Patient characteristics

Table 1 presents the baseline characteristics according to sex and VFA category. The mean age at baseline was 59.2 ± 12.8 years. The 200 participants (107 men, 93 women) comprised 71 patients with VFA < 100 cm^2^ and 129 patients with VFA ≥ 100 cm^2^. Patients with VFA ≥ 100 cm^2^ had a higher V/S ratio (0.97 ± 0.46 vs. 0.58 ± 0.41) and lower eGFR (53.4 ± 22.7 vs. 60.7 ± 21.3 mL/min/1.73 m^2^) than did patients with VFA < 100 cm^2^. Additionally, male patients had a higher V/S ratio (1.08 ± 0.45 vs. 0.54 ± 0.32) and lower eGFR (52.8 ± 22.5 vs. 59.7 ± 21.9 mL/min/1.73 m^2^) than did female patients. The median follow-up duration was 12.8 years (interquartile range: 5.9–12.9 years), during which 8 patients died, 47 were lost to follow up, and 84 reached the end-point (i.e., ≥30% eGFR decline or ESRD).

**Table 1:** Patient characteristics according to sex and visceral fat area category (*n* = 200).

Variables	Entire Cohort *n* = 200	Visceral fat area <100 cm^2^	Visceral fat area ≥100 cm^2^	*P*-Value	Men	Women	*P*-Value
		*n* = 71	*n* = 129		*n* = 107	*n* = 93	
*Clinical and Laboratory Findings*							
Age (years)	59.2 ± 12.8 [200]	54.6 ± 13.1	61.7 ± 12.0	0.0001	59.7 ± 12.9	58.6 ± 12.8	0.5448
Sex (Men; %)	107 (53.5) [200]	21 (29.6)	86 (66.7)	<0.0001	107 (100.0)	0 (0.0)	<0.0001
MBP (mmHg)	92.6 ± 6.3 [200]	89.7 ± 5.8	94.2 ± 6.0	<0.0001	93.4 ± 6.3	91.7 ± 6.3	0.0491
PP (mmHg)	48.8 ± 4.1 [200]	47.6 ± 3.4	49.4 ± 4.4	0.0023	49.3 ± 4.7	48.2 ± 3.3	0.0794
BMI (kg/m^2^)	24.0 ± 3.9 [200]	21.4 ± 3.0	25.4 ± 3.6	<0.0001	24.6 ± 3.4	23.4 ± 4.3	0.0342
Visceral fat area (cm^2^)	126.9 ± 61.4 [200]	64.0 ± 25.1	161.4 ± 46.1	<0.0001	150.1 ± 60.5	100.1 ± 50.8	<0.0001
Subcutaneous fat area (cm^2^)	176.5 ± 89.1 [200]	141.0 ± 78.4	196.1 ± 88.9	<0.0001	152.1 ± 69.9	204.6 ± 100.2	<0.0001
V/S ratio	0.83 ± 0.48 [200]	0.58 ± 0.41	0.97 ± 0.46	<0.0001	1.08 ± 0.45	0.54 ± 0.32	<0.0001
V/S ratio ≥0.25 (vs. no)	191 (95.5) [200]	62 (87.3)	129 (100.0)	<0.0001	107 (100.0)	84 (90.3)	0.0008
V/S ratio ≥0.3 (vs. no)	186 (93.0) [200]	58 (81.7)	128 (99.2)	<0.0001	107 (100.0)	79 (85.0)	<0.0001
V/S ratio ≥0.5 (vs. no)	149 (74.5) [200]	37 (52.1)	112 (86.8)	<0.0001	101 (94.4)	48 (51.6)	<0.0001
V/S ratio ≥0.55 (vs. no)	134 (67.0) [200]	26 (36.6)	108 (83.7)	<0.0001	99 (92.5)	35 (37.6)	<0.0001
V/S ratio ≥0.7 (vs. no)	103 (51.5) [200]	17 (23.9)	86 (66.7)	<0.0001	83 (77.6)	20 (21.5)	<0.0001
V/S ratio ≥0.75 (vs. no)	85 (42.5) [200]	12 (16.9)	73 (56.6)	<0.0001	74 (69.2)	11 (11.8)	<0.0001
V/S ratio ≥1.0 (vs. no)	56 (28.0) [200]	6 (8.5)	50 (38.8)	<0.0001	53 (49.5)	3 (3.2)	<0.0001
V/S ratio ≥1.25 (vs. no)	34 (17.0) [200]	4 (5.6)	30 (23.3)	0.0015	33 (30.8)	1 (1.1)	<0.0001
eGFR (mL/min/1.73m^2^)	56.0 ± 22.4 [200]	60.7 ± 21.3	53.4 ± 22.7	0.0258	52.8 ± 22.5	59.7 ± 21.9	0.0299
UACR (mg/g Cre)	66.2 (22.3–252.5) [200]	63.5 (21.3–150.4)	70.1 (23.3–554.6)	0.3364	90.2 (26.0–860.2)	50.9 (21.1–115.6)	0.0153
*Primary cause of CKD*							
Diabetic nephropathy (%)	18 (9.0) [200]	3 (4.2)	15 (11.6)	0.1196	12 (11.2)	6 (6.5)	0.3233
Chronic glomerulonephritis (%)	104 (52.0) [200]	49 (69.0)	55 (42.6)	0.0004	47 (43.9)	57 (61.3)	0.0142
Nephrosclerosis (%)	41 (20.5) [200]	4 (5.6)	37 (28.7)	0.0001	33 (30.8)	8 (8.6)	0.0001
Others (%)	37 (18.5) [200]	15 (21.1)	22 (17.1)	0.4779	15 (14.0)	22 (23.7)	0.0800
*Concomitant drugs*							
Antihypertensive agents (%)	140 (70.0) [200]	45 (63.4)	95 (73.6)	0.1296	84 (78.5)	56 (60.2)	0.0049
ARB and or ACEI	113 (56.5) [200]	34 (47.9)	79 (61.2)	0.0683	72 (67.3)	41 (44.1)	0.0010
CCB	62 (31.0) [200]	15 (21.1)	47 (36.4)	0.0251	34 (31.8)	28 (30.1)	0.7992
Antihyperuricemic agents (%)	78 (39.0) [200]	17 (23.9)	61 (47.3)	0.0012	60 (56.1)	18 (19.4)	<0.0001
Antidiabetic agents (%)	26 (13.0) [200]	6 (8.5)	20 (15.5)	0.1905	17 (15.9)	9 (9.7)	0.1927
Corticosteroids (%)	28 (14.0) [200]	13 (18.3)	15 (11.6)	0.2066	16 (15.0)	12 (12.9)	0.6769
Immunosuppressants (%)	13 (6.5) [200]	4 (5.6)	9 (7.0)	1.0000	8 (7.5)	5 (5.4)	0.5811
Diuretics (%)	51 (25.5) [200]	17 (23.9)	34 (26.4)	0.7079	25 (23.4)	26 (28.0)	0.4573
*Comorbidities*							
Hypertension (%)	139 (69.5) [200]	45 (63.4)	94 (72.9)	0.1631	83 (77.6)	56 (60.2)	0.0078
Hyperuricemia (%)	100 (50.0) [200]	21 (29.6)	79 (61.2)	<0.0001	75 (70.1)	25 (26.9)	<0.0001
Hypertriglyceridemia (%)	143 (71.5) [200]	41 (57.8)	102 (79.1)	0.0014	83 (77.6)	60 (64.5)	0.0414
Hypercholesterolemia (%)	123 (61.5) [200]	38 (53.5)	85 (65.9)	0.0854	63 (58.9)	60 (64.5)	0.4138
Low HDL cholesterol (%)	93 (46.5) [200]	24 (33.8)	69 (53.5)	0.0076	56 (52.3)	37 (39.8)	0.0759
Hyperglycemia (%)	66 (33.0) [200]	16 (22.5)	50 (38.8)	0.0195	43 (40.2)	23 (24.7)	0.0204
Diabetes mellitus (%)	42 (21.0) [200]	8 (11.3)	34 (26.4)	0.0122	25 (23.4)	17 (18.3)	0.3785

Continuous variables are expressed as means and standard deviations or as medians (quartile 1, quartile 3). Categorical variables are expressed as n (%). Values of nonmissing data are shown in []. Abbreviations: *n*, number; *P*, calculated probability; CKD, chronic kidney disease; MBP, mean blood pressure; PP, pulse pressure; BMI, body mass index; eGFR, estimated glomerular filtration rate; UACR, urine albumin-to-creatinine ratio; ARB, angiotensin Ⅱ receptor blocker; ACEI, angiotensin-converting enzyme inhibitor; CCB, calcium-channel blocker; V/S visceral to subcutaneous fat ratio; Cre, creatine.

### V/S ratio as a progression-related factor in patients with chronic kidney disease

**Fig 1:**
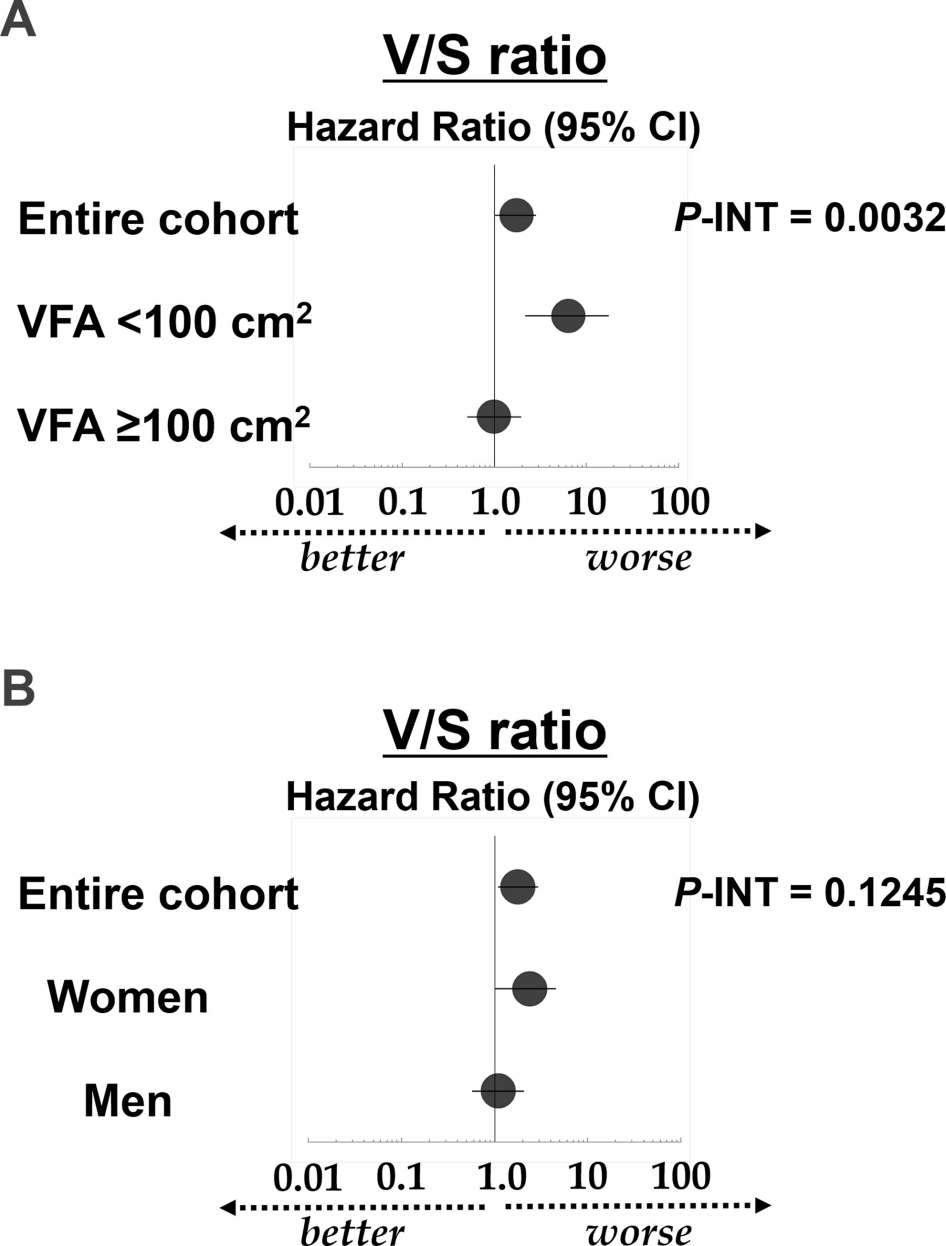
Hazard ratios of the V/S ratio for the renal prognosis derived from the multivariate Cox proportional hazards analyses according to VFA category (A) and sex (B). The circles represent HRs and the bars represent 95% CIs for the association with the renal prognosis, with an eGFR decline ≥30% or end-stage renal disease as the endpoint (derived from Tables 2 and 3). The *P*-value for the interaction is derived from Table 2. Abbreviations: V/S ratio, visceral to subcutaneous fat ratio; VFA, visceral fat area; HR, hazard ratio; CI, confidence interval; eGFR, estimated glomerular filtration rate; *P*, calculated probability; *P*-INT, *P*-value for the interaction.

Two multivariate Cox analyses of the renal prognosis were performed, each with interaction terms for sex or VFA category (Table 2). The V/S ratio was revealed as a significant renal prognostic factor in both multivariate Cox analyses. Additionally, there was a significant interaction between the VFA category and the V/S ratio in terms of the risk of CKD progression. When stratified by the VFA category, the hazard ratio (HR) of the V/S ratio was higher in the sub-cohort of patients with VFA **<**100 cm^2^ than in the sub-cohort of patients with VFA ≥ 100 cm^2^ (HR: 6.42 vs. 1.00) (Table 3). Additionally, when the V/S ratio variable was removed from the model and replaced by the VFA (10-cm^2^ increase), with all other variables fixed, the VFA (10-cm^2^ increase) failed to demonstrate a statistical association with renal outcomes in patients with VFA **<**100 cm^2^ (Table 3). In contrast, sex did not significantly interact with the V/S ratio in terms of the renal prognosis; however, when stratified by sex, there was a significant association between the V/S ratio and renal prognosis in women, but not in men (HR: 2.40 vs. 1.10) (Table 3). Fig 1 shows the hazard ratios of the V/S ratio for the renal prognosis derived from the multivariate Cox proportional hazards analyses according to the VFA category (Fig 1A) and sex (Fig 1B).

**Table 2:** Results of the univariate and multivariate analyses for the risk factors associated with renal outcomes (i.e., a ≥ 30% estimated glomerular filtration rate decline or end-stage renal disease) among the entire study population (*n* = 200).

Variables	Univariate Analysis		Multivariate Analysis with VFA interaction terms			Multivariate Analysis with sex interaction terms		
	Hazard Ratio (95% CI)	*P*-Value	Hazard Ratio (95% CI)	*P*-Value	*P*-INT with VFA ≥100 cm^2^	Hazard Ratio (95% CI)	*P*-Value	*P*-INT with sex
Age (1-year increase)	1.02 (1.00–1.04)	0.0440	0.98 (0.96–1.01)	0.1268	0.2546	0.98 (0.96–1.01)	0.1708	0.9724
Men (vs. women)	1.45 (0.95–2.25)	0.0881	0.79 (0.43–1.46)	0.4597	0.4288	0.63 (0.34–1.18)	0.1493	-
eGFR (10-mL/min/1.73 m^2^ increase)	0.63 (0.55–0.71)	<0.0001	0.64 (0.55–0.75)	<0.0001	0.0317	0.67 (0.58–0.77)	<0.0001	0.9868
PP (10-mmHg increase)	3.05 (1.90–4.70)	<0.0001	2.90 (1.62–5.18)	0.0003	-	2.80 (1.56–4.89)	0.0004	-
UACR (10-mg/g Cre increase)	1.00 (1.00–1.01)	<0.0001	1.00 (1.00–1.01)	0.0190	-	1.00 (1.00–1.01)	0.0306	-
VFA (10-cm^2^ increase)	1.07 (1.03–1.10)	0.0004	-	-	-	-	-	-
SFA (10-cm^2^ increase)	1.01 (0.99–1.04)	0.2773	-	-	-	-	-	-
V/S ratio	1.58 (1.08–2.23)	0.0209	1.78 (1.05–3.01)	0.0317	0.0032	1.76 (1.01–2.86)	0.0292	0.1245
Hypertension (vs. no)	2.16 (1.30–3.79)	0.0024	0.95 (0.39–2.29)	0.9026	-	1.00 (0.40–2.33)	0.9953	-
Hyperuricemia (vs. no)	3.28 (2.09–5.27)	<0.0001	1.53 (0.84–2.79)	0.1660	-	1.54 (0.86–2.84)	0.1566	-
Low HDL-C (vs. no)	1.76 (1.15–2.74)	0.0097	1.30 (0.78–2.16)	0.3193	-	1.16 (0.71–1.90)	0.5613	-
Hyperglycemia (vs. no)	1.95 (1.25–3.01)	0.0035	-	-	-	-	-	-
Diabetes mellitus (vs. no)	2.62 (1.62–4.12)	<0.0001	1.49 (0.85–2.61)	0.1657	-	1.71 (0.96–2.97)	0.0615	-
ARB and or ACEI use (vs. no)	2.13 (1.36–3.44)	0.0013	1.22 (0.54–2.75)	0.6235	-	1.16 (0.55–2.74)	0.7082	-

The V/S ratio, as well as age, sex, eGFR, and interaction terms (age, sex, eGFR, and V/S ratio * sex/ VFA ≥100 cm^2^), were included in the multivariate model. The *P*-value for the interaction corresponds to the interaction between sex or VFA category and each variable of interest, in terms of the renal outcomes, in the multivariate analysis. Abbreviations: *n*, number; CI, confidence interval; *P*, calculated probability; *P*-INT, *P*-value for interaction; VFA, visceral fat area; vs, versus; eGFR, estimated glomerular filtration rate; PP, pulse pressure; UACR, urine albumin-to-creatinine ratio; V/S ratio, visceral to subcutaneous fat ratio; SFA, subcutaneous fat area; HDL-C, high-density lipoprotein cholesterol; ARB, angiotensin Ⅱ receptor blocker; ACEI, angiotensin-converting enzyme inhibitor.

**Table 3:** Results of the multivariate analyses for the risk factors associated with disease progression (i.e., a ≥30% estimated glomerular filtration rate decline or end-stage renal disease) according to sex or VFA category.

Variables	VFA <100 cm^2^ for the V/S ratio (*n* = 71)		VFA <100 cm^2^ for VFA (*n* = 71)		VFA ≥100 cm^2^ (*n* = 129)		Men (*n* = 107)		Women (*n* = 93)	
	Hazard Ratio (95% CI)	*P*-Value	Hazard Ratio (95% CI)	*P*-Value	Hazard Ratio (95% CI)	*P*-Value	Hazard Ratio (95% CI)	*P*-Value	Hazard Ratio (95% CI)	*P*- Value
Age (1-year increase)	0.95 (0.91–1.00)	0.0370	0.98 (0.94–1.02)	0.3149	0.99 (0.96–1.02)	0.4369	0.98 (0.94–1.02)	0.1874	0.98 (0.95–1.02)	0.3071
Men (vs. women)	0.60 (0.16–2.26)	0.4270	0.64 (0.16–2.57)	0.5159	0.93 (0.46–1.87)	0.8462	-	-	-	-
eGFR (10-mL/min/1.73 m^2^ increase)	0.41 (0.28–0.60)	<0.0001	0.50 (0.37–0.68)	<0.0001	0.69 (0.60–0.80)	<0.0001	0.64 (0.52–0.77)	<0.0001	0.62 (0.51–0.75)	<0.0001
PP (10-mmHg increase)	18.65 (3.24–107.24)	0.0008	8.85 (1.75–44.81)	0.0069	3.08 (1.76–5.41)	<0.0001	3.62 (2.00–6.28)	<0.0001	2.12 (0.62–7.42)	0.2313
UACR (10-mg/g Cre increase)	1.04 (1.02–1.06)	<0.0001	1.04 (1.02–1.05)	<0.0001	1.00 (1.00–1.00)	0.0133	1.00 (1.00–1.00)	0.0295	1.01 (1.00–1.02)	0.0421
VFA (10-cm^2^ increase)	-	-	0.92 (0.75–1.13)	0.4288	-	-	-	-	-	-
V/S ratio	6.42 (2.39–17.25)	0.0023	-	-	1.00 (0.51–1.96)	0.9970	1.10 (0.57–2.06)	0.7636	2.40 (1.02–4.57)	0.0457

Variables with *P*-INT <0.05 in Table 2, as well as the V/S ratio, VFA (10-cm^2^ increase), age, sex, and eGFR, were included in the multivariate models. Abbreviations: *n*, number; CI, confidence interval; *P*, calculated probability; VFA, visceral fat area; vs., versus; eGFR, estimated glomerular filtration rate; PP, pulse pressure; UACR, urine albumin-to-creatinine ratio; V/S ratio, visceral to subcutaneous fat ratio; Cre, creatine.

### Time-series changes in pseudo-*R^2^* Values in terms of the prognostic efficacy

**Fig 2:**
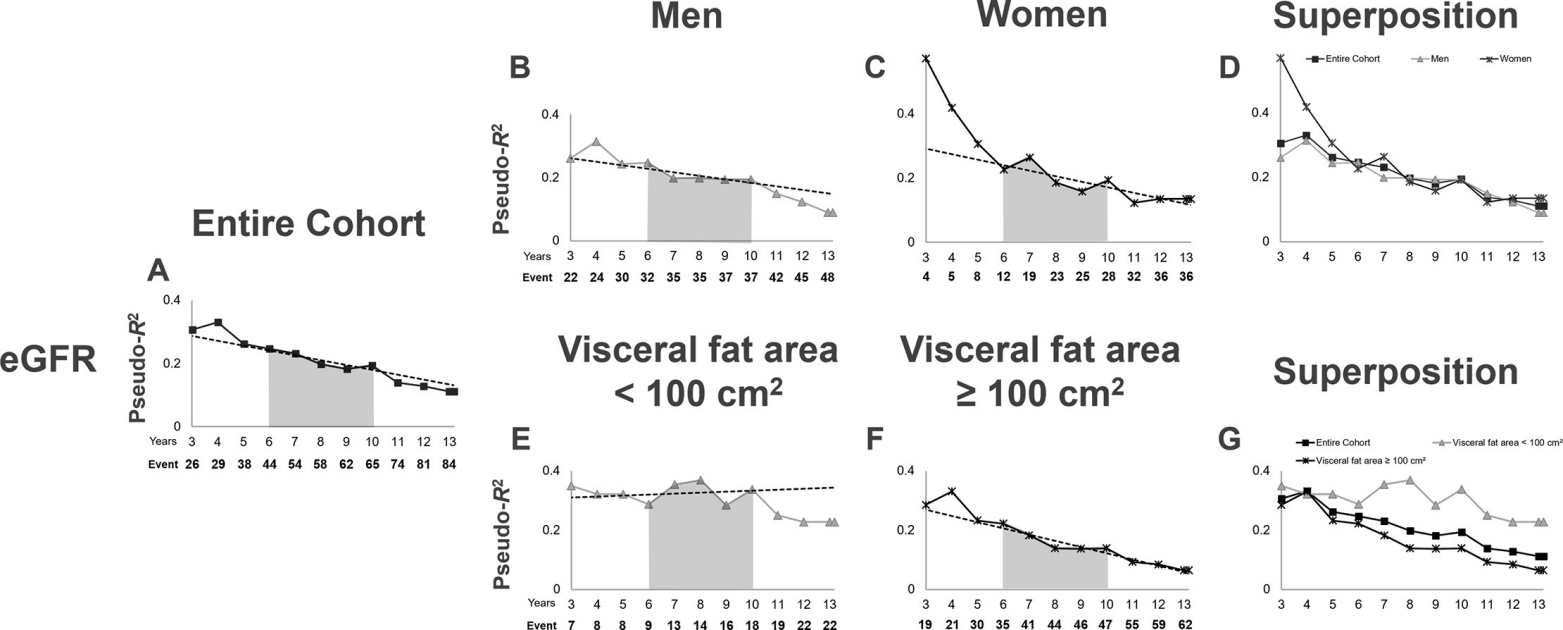
Time-series changes in the pseudo-*R*^2^ values for the renal outcome in terms of the eGFR. Pseudo-*R*^2^ values for the entire cohort (A), men (B), women (C), patients with VFA <100 cm^2^ (E), and patients with VFA ≥100 cm^2^ (F). The lines represent the time-series changes in the pseudo-*R*^2^ values for the renal outcome in terms of the eGFR. Dotted lines mark the least-squares regression line from the 6^th^ year to the 10^th^ year. The gray area under the lines represents the 6Y–10Y Mean. (A) Pseudo-*R*^2^ values for all cohorts are presented superimposed on those for each sex (D) and VFA category (G). The lines represent the time-series changes in the pseudo-*R*^2^ values for the renal outcome in terms of the eGFR for the entire cohort, men, women, patients with VFA <100 cm^2^, and patients with VFA ≥100 cm^2^ (shown individually in A–C, E, and F). Abbreviations: eGFR, estimated glomerular filtration rate; VFA, visceral fat area; 6Y–10Y Mean, the mean value of the pseudo-*R*^2^ values from the 6^th^ year to the 10^th^ year.

Initially, we examined the pseudo-*R*^2^ values for the eGFR, which is an established potent risk factor for CKD progression (Table 4, Fig 2). As the pseudo-*R*^2^ values for the eGFR were comparable between men and women from year 6 to the end of the study (Fig 2D), we determined that the pseudo-*R*^2^ values after the 6^th^ year were reliable and universal. In order to easily compare our results with those of future studies, we attempted to establish indicators of the 6^th^ to 10^th^ years as indexes that would not be influenced by the follow-up periods in future studies. Accordingly, we calculated the mean value and percent change of the pseudo-*R*^2^ values for the 6^th^ year to the 10^th^ year (6Y–10Y Mean and 6Y-10Y Change, respectively). Interestingly, unlike the comparison between men and women, there was a difference in the time-series changes in pseudo-*R*^2^ values for the eGFR from the 6^th^ year to the 10^th^ year between patients with VFA <100 cm^2^ and patients with VFA ≥100 cm^2^. Specifically, the 6Y–10Y Mean for eGFR was clearly higher in patients with VFA <100 cm^2^ than in patients with VFA ≥100 cm^2^ (Fig 2, shown by the gray area under the lines in Fig 2E and 2F; Table 4, 6Y–10Y Mean of VFA <100 cm^2^ vs. VFA ≥100 cm^2^: 0.3301 vs. 0.1603). The pseudo-R^2^ values for the eGFR in patients with VFA <100 cm^2^ showed an almost flat (slightly increasing) pattern from the 6^th^ year to the 10^th^ year, with a 6Y–10Y Change of 1.1 (%/year) (Table 4, Fig 2E). In contrast, there was a decreasing pattern in patients with VFA ≥100 cm^2^, with a 6Y–10Y Change of -9.5 (%/year) (Table 4, Fig 2F), suggesting the existence of renal pathophysiological differences between patients with and without VFA ≥100 cm^2^.

**Table 4:** Time-series changes in pseudo-*R*^2^ values of the prognostic efficacy for renal outcomes: eGFR (*n* = 200).

Cohort	Entire Cohort	VFA <100 cm^2^	VFA ≥100 cm^2^	Men	Women
*Years/Period*	eGFR	eGFR	eGFR	eGFR	eGFR
**1Y**	0.3891	0.5172	0.4189	0.2974	1.0000
**2Y**	0.2882	0.6423	0.2249	0.2762	1.0000
**3Y**	0.3065	0.3498	0.2856	0.2613	0.5731
**4Y**	0.3314	0.3212	0.3311	0.3146	0.4192
**5Y**	0.2626	0.3212	0.2331	0.2439	0.3067
**6Y**	0.2470	0.2871	0.2222	0.2468	0.2268
**7Y**	0.2315	0.3542	0.1830	0.1986	0.2638
**8Y**	0.1978	0.3690	0.1393	0.1986	0.1857
**9Y**	0.1821	0.2844	0.1381	0.1937	0.1585
**10Y**	0.1945	0.3384	0.1394	0.1937	0.1935
**11Y**	0.1388	0.2503	0.0929	0.1504	0.1227
**12Y**	0.1283	0.2277	0.0849	0.1226	0.1351
**13Y**	0.1116	0.2277	0.0651	0.0899	0.1351
**END**	0.1116	0.2277	0.0651	0.0899	0.1351
**6Y–10Y Mean**	0.2080	0.3301	0.1603	0.2028	0.2045
**6Y–10Y Change (%/year)**	-6.3	1.1	-9.5	-4.5	-7.6

Abbreviations: eGFR, estimated glomerular filtration rate; VFA, visceral fat area; Y, years; End, study end; 6–10Y Mean, the mean value of the pseudo-*R*^2^ during years 6 to 10; 6Y–10Y Change, the % change of pseudo-*R*^2^ values from the 6^th^ year to the 10^th^ year.

**Fig 3:**
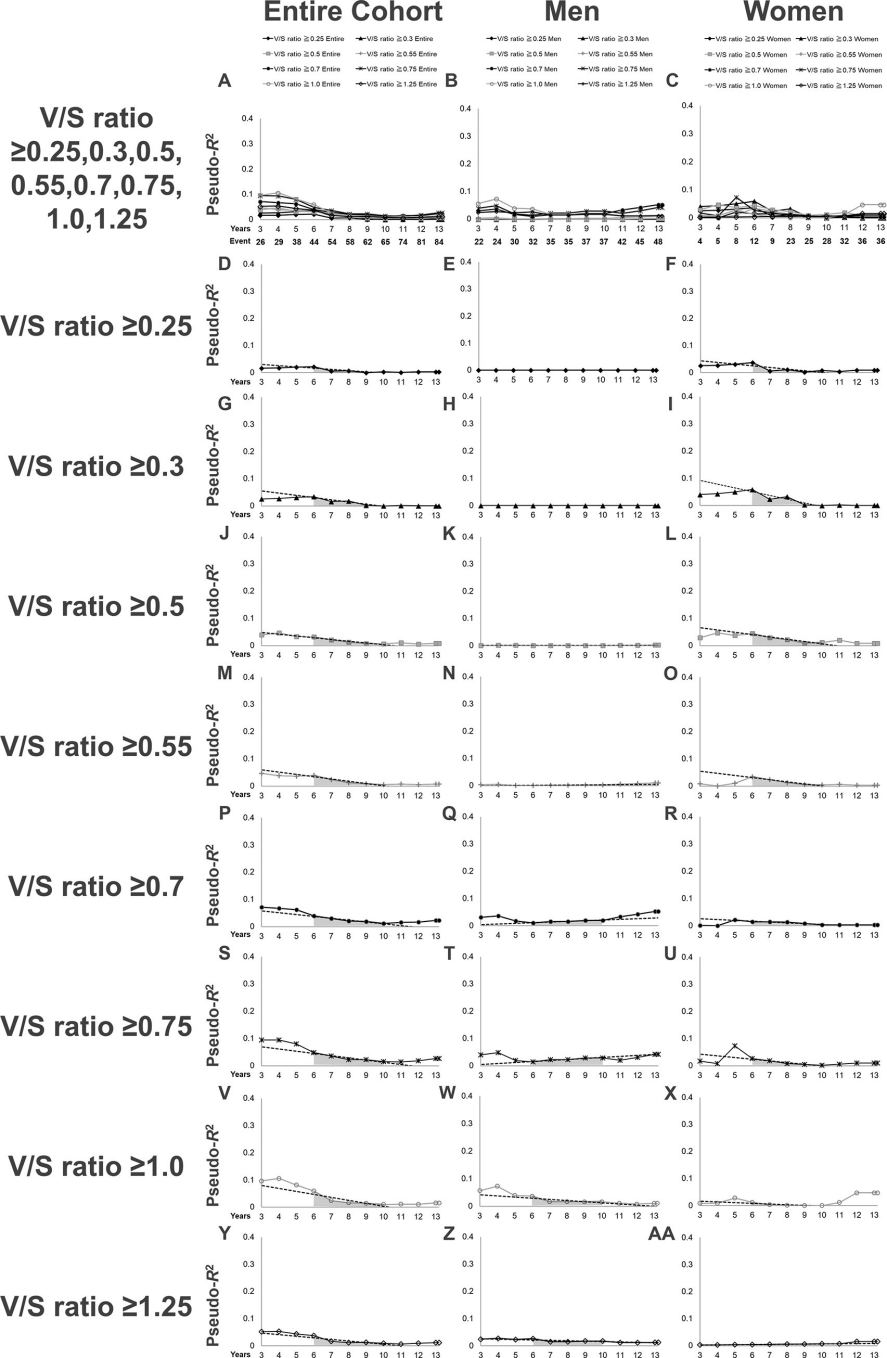
Time-series changes in the pseudo-*R*^2^ values for the renal outcome in terms of each definition of a V/S ratio in the entire cohort. (A-C): Pseudo-*R*^2^ values for all definitions of a high V/S ratio for the entire cohort and each sex are presented superimposed. The lines represent the time-series changes in the pseudo-*R*^2^ values for the renal outcome in terms of each definition of a high V/S ratio in the entire cohort (A), men (B), and women (C). (D–F): Pseudo-*R*^2^ values in terms of a V/S ratio ≥0.25. (G–I): Pseudo-*R*^2^ values in terms of a V/S ratio ≥0.3. (J–L): Pseudo-*R*^2^ values in terms of a V/S ratio ≥0.5. (M–O): Pseudo-*R*^2^ values in terms of a V/S ratio ≥0.55. (P–R): Pseudo-*R*^2^ values in terms of a V/S ratio ≥0.7. (S–U): Pseudo-*R*^2^ values in terms of a V/S ratio ≥0.75. (V–X): Pseudo-*R*^2^ values in terms of a V/S ratio ≥1.0. (Y–AA): Pseudo-*R*^2^ values in terms of a V/S ratio ≥1.25. Dotted lines mark the least-squares regression line from the 6^th^ year to the 10^th^ year. The gray area under the lines represents the 6Y–10Y Mean. Abbreviations: V/S ratio, visceral to subcutaneous fat ratio; 6Y–10Y Mean, the mean value of the pseudo-*R*^2^ values from the 6^th^ year to the 10^th^ year.

Second, we examined the pseudo-*R*^2^ values for the renal outcome in terms of different cut-off points for defining a high V/S ratio (Table 5, Fig 3). For all eight cut-off points of the V/S ratio examined, the pseudo-*R*^2^ values in the entire cohort gradually fell over time (Fig 3A, 3D, 3G, 3J, 3M, 3P, 3S, 3V and 3Y). The highest value of the 6Y–10Y Mean in the entire cohort (0.0281) was obtained with a V/S ratio ≥0.75. When stratified by sex, the highest value of the 6Y–10Y Mean in men (0.0236) was obtained with a V/S ratio ≥0.75, and the highest value of the 6Y–10Y Mean in women (0.0221) was obtained with a V/S ratio ≥0.5. Importantly, the pseudo-*R*^2^ values for a V/S ratio ≥0.7 in men showed an increasing pattern after 6 years, with a 6Y–10Y Change of 25.1 (%/year), and rose to its highest value (0.0522) at the end of the follow-up period (Table 4, Fig 3Q), at which point, the pseudo-*R*^2^ value for a V/S ratio ≥0.7 was even higher than that for a V/S ratio ≥0.75, suggesting that men with a V/S ratio ≥0.7 might have a poor renal prognosis with longer follow-up.

**Table 5:** Time-series changes in pseudo-*R*^2^ values of the prognostic efficacy for renal outcomes in terms of the V/S ratio in the entire cohort.

*Years/Period*	V/S ratio ≥0.25	V/S ratio ≥0.30	V/S ratio ≥0.50	V/S ratio ≥0.55	V/S ratio ≥0.70	V/S ratio ≥0.75	V/S ratio ≥1.0	V/S ratio ≥1.25
**A, Entire Cohort (n = 200)**								
**1Y**	0.0095	0.0150	0.0608	0.0827	0.0242	0.0453	0.0991	**0.1710**
**2Y**	0.0141	0.0222	0.0906	0.1236	0.1395	**0.1456**	0.1264	0.1109
**3Y**	0.0166	0.0262	0.0397	0.0471	0.0711	0.0950	**0.0965**	0.0524
**4Y**	0.0175	0.0275	0.0464	0.0388	0.0674	0.0943	**0.1058**	0.0533
**5Y**	0.0200	0.0316	0.0329	0.0358	0.0623	0.0806	**0.0814**	0.0441
**6Y**	0.0218	0.0344	0.0319	0.0389	0.0395	0.0486	**0.0598**	0.0371
**7Y**	0.0061	0.0163	0.0206	0.0242	0.0297	**0.0362**	0.0245	0.0167
**8Y**	0.0072	0.0183	0.0128	0.0125	0.0207	**0.0221**	0.0161	0.0116
**9Y**	0.0001	0.0028	0.0075	0.0087	0.0191	**0.0225**	0.0145	0.0127
**10Y**	0.0023	0.0004	0.0063	0.0049	0.0111	**0.0149**	0.0101	0.0096
**11Y**	0.0008	0.0018	0.0105	0.0073	**0.0156**	0.0142	0.0111	0.0066
**12Y**	0.0032	0.0005	0.0055	0.0049	0.0164	**0.0180**	0.0107	0.0096
**13Y**	0.0026	0.0009	0.0079	0.0077	0.0232	**0.0267**	0.0156	0.0118
**End**	0.0026	0.0009	0.0079	0.0077	0.0232	**0.0267**	0.0156	0.0118
**6Y–10Y Mean**	0.0064	0.0137	0.0150	0.0168	0.0237	**0.0281**	0.0225	0.0161
**6Y**–**10Y Change (%/year)**	-20.6	-23.7	-20.2	-21.5	-17.1	-16.7	-18.3	**-15.9**
**B, Men (*n* = 107)**								
**1Y**	0.0000	-0.0000	0.012	0.0173	0.0566	0.0823	0.1573	**0.2651**
**2Y**	-0.0000	-0.0000	0.0222	0.0299	**0.0987**	0.0744	0.0561	0.0585
**3Y**	0.0000	0.0000	0.0006	0.0035	0.0303	0.0397	**0.0564**	0.0243
**4Y**	0.0000	0.0000	0.0011	0.0049	0.0364	0.0485	**0.0730**	0.0273
**5Y**	-0.0000	-0.0000	0.0007	0.0003	0.0169	0.0191	**0.0391**	0.0232
**6Y**	0.0000	0.0000	0.0003	0.0008	0.0098	0.0137	**0.0367**	0.0265
**7Y**	0.0000	0.0000	0.0000	0.0018	0.0155	**0.0222**	0.0169	0.0148
**8Y**	0.0000	0.0000	0.0000	0.0018	0.0155	**0.0222**	0.0169	0.0148
**9Y**	0.0000	0.0000	0.0000	0.0027	0.0199	**0.0287**	0.0162	0.0177
**10Y**	0.0000	0.0000	0.0000	0.0027	0.0199	**0.0287**	0.0162	0.0177
**11Y**	0.0000	0.0000	0.0007	0.0054	**0.0328**	0.0206	0.0112	0.0118
**12Y**	0.0000	0.0000	0.0014	0.0075	**0.0419**	0.0303	0.0078	0.0120
**13Y**	0.0000	0.0000	0.0024	0.0099	**0.0522**	0.0418	0.0107	0.0123
**End**	0.0000	0.0000	0.0024	0.0099	**0.0522**	0.0418	0.0107	0.0123
**6Y–10Y Mean**	0.0000	0.0000	0.0000	0.0020	0.0164	**0.0236**	0.0191	0.0174
**6Y–10Y Change (%/year)**	NA	NA	-20.0	**58.8**	25.1	26.6	-11.4	-5.5
**C, Women (n = 93)**								
**1Y**	0.0185	0.0297	0.1206	**0.1784**	0.0441	0.0229	0.0060	0.0020
**2Y**	0.0185	0.0297	0.1206	**0.1784**	0.0441	0.0229	0.0060	0.0020
**3Y**	0.0253	**0.0405**	0.0291	0.0080	0.0009	0.0169	0.0081	0.0027
**4Y**	0.0269	0.0432	**0.0470**	0.0003	0.0002	0.0075	0.0087	0.0029
**5Y**	0.0313	0.0502	0.0368	0.0102	0.0215	**0.0731**	0.0280	0.0033
**6Y**	0.0367	**0.0590**	0.0441	0.0340	0.0146	0.0264	0.0121	0.0039
**7Y**	0.0064	0.0231	**0.0293**	0.0237	0.0142	0.0183	0.0030	0.0049
**8Y**	0.0111	**0.0325**	0.0221	0.0128	0.0131	0.0081	0.0011	0.0055
**9Y**	0.0019	0.0024	**0.0089**	0.0054	0.0076	0.0050	0.0006	0.0058
**10Y**	0.0080	0.0002	**0.0118**	0.0041	0.0025	0.0020	0.0001	0.0063
**11Y**	0.0036	0.0021	**0.0195**	0.0064	0.0029	0.0054	0.0112	0.0071
**12Y**	0.0093	0.0005	0.0086	0.0033	0.0034	0.0103	**0.0472**	0.0154
**13Y**	0.0093	0.0005	0.0086	0.0033	0.0034	0.0103	**0.0472**	0.0154
**End**	0.0093	0.0005	0.0086	0.0033	0.0034	0.0103	**0.0472**	0.0154
**6Y–10Y Mean**	0.0104	0.0219	**0.0221**	0.0152	0.0109	0.0114	0.0027	0.0053
**6Y–10Y Change (%/year)**	-16.9	-23.4	-19.3	-23.0	-21.1	-23.5	-21.8	**14.6**

The pseudo-*R*^2^ values in bold identify the highest values among the various V/S ratios within the same year. The values of 6Y–10Y Mean and 6Y–10Y Change in bold identify the highest values among the various V/S ratios within the same period. Abbreviations: V/S ratio, visceral to subcutaneous fat ratio; Y, years; 6–10Y Mean, the mean value of the pseudo-*R*^2^ during years 6 to 10; 6Y–10Y Change, the % change of pseudo-*R*^2^ values from the 6^th^ year to the 10^th^ year.

**Fig 4:**
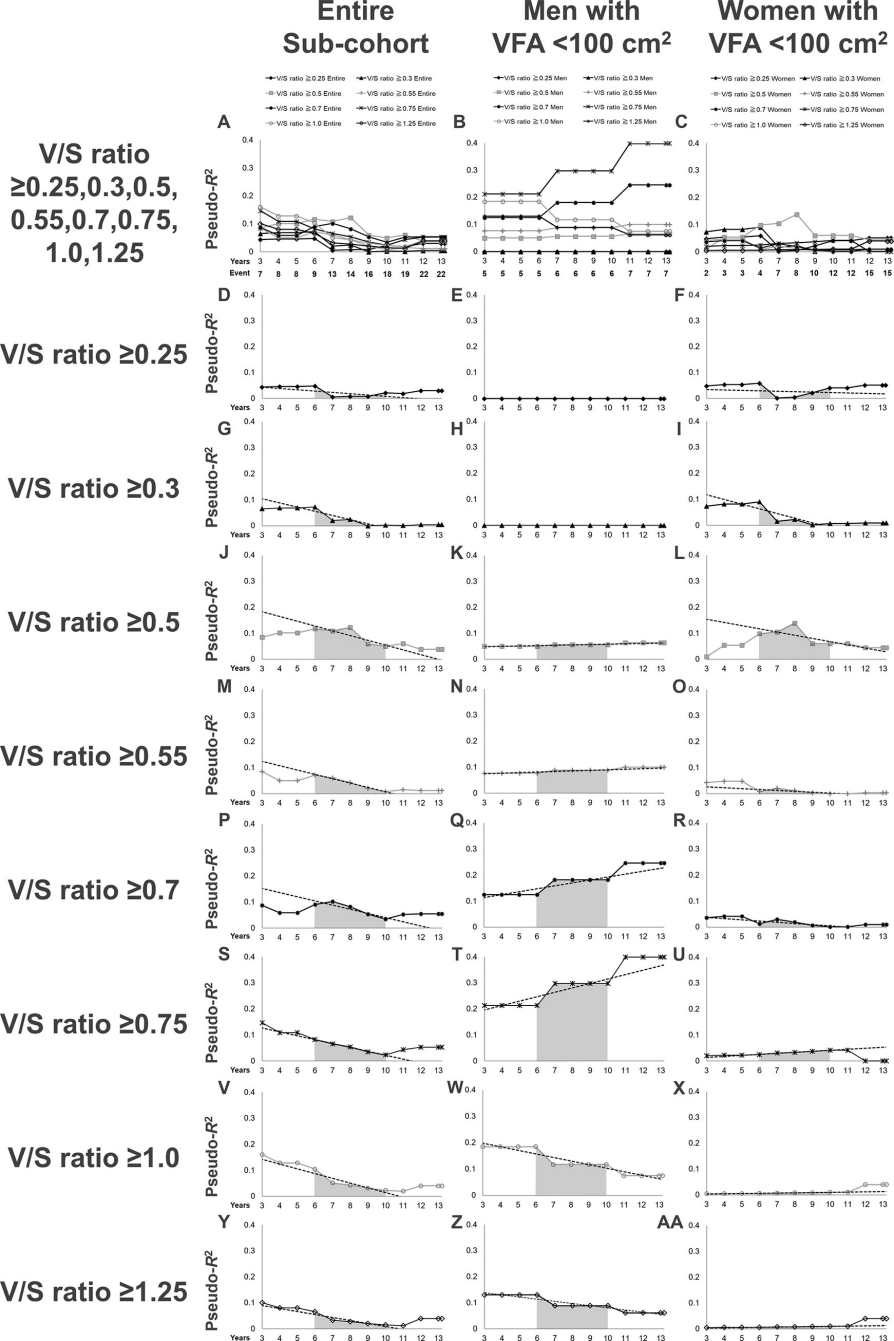
Time-series changes in the pseudo-*R*^2^ values for the renal outcome in terms of each definition of a high V/S ratio in the sub-cohort of patients with VFA <100 cm^2^. (A-C): Pseudo-*R*^2^ values for all definitions of a high V/S ratio for the entire sub-cohort and each sex are presented superimposed. The lines represent the time-series changes in the pseudo-*R*^2^ values for the renal outcome in terms of each definition of a high V/S ratio for the entire sub-cohort (A), the sub-cohort of men with VFA <100 cm^2^ (B), and the sub-cohort of women with VFA <100 cm^2^ (C). (D–F): Pseudo-*R*^2^ values in terms of a V/S ratio ≥0.25. (G–I): Pseudo-*R*^2^ values in terms of a V/S ratio ≥0.3. (J–L): Pseudo-*R*^2^ values in terms of a V/S ratio ≥0.5. (M–O): Pseudo-*R*^2^ values in terms of a V/S ratio ≥0.55. (P–R): Pseudo-*R*^2^ values in terms of a V/S ratio ≥0.7. (S–U): Pseudo-*R*^2^ values in terms of a V/S ratio ≥0.75. (V–X): Pseudo-*R*^2^ values in terms of a V/S ratio ≥1.0. (Y–AA): Pseudo-*R*^2^ values in terms of a V/S ratio ≥1.25. Dotted lines mark the least-squares regression line from the 6^th^ year to the 10^th^ year. The gray area under the lines represents the 6Y–10Y Mean. Abbreviations: V/S ratio, visceral to subcutaneous fat ratio; 6Y–10Y Mean, the mean value of the pseudo-*R*^2^ values from the 6^th^ year to the 10^th^ year.

Third, considering the observation of a significant interaction with VFA category in the multivariate Cox regression analysis, we also examined the pseudo-*R*^2^ values for the renal outcome in terms of different cut-off points for defining a high V/S ratio in the sub-cohort of patients with VFA <100 cm^2^ (Fig 4). The highest value of the 6Y–10Y Mean in patients with VFA <100 cm^2^ (0.0937) was obtained with a V/S ratio ≥0.5. When stratified by sex, the highest value of the 6Y–10Y Mean in men (0.2875) was obtained with a V/S ratio ≥0.75, and the highest value of the 6Y–10Y Mean in women (0.0959) was obtained with a V/S ratio ≥0.5. Importantly, the pseudo-*R*^2^ values for a V/S ratio ≥0.5 in men with VFA <100 cm^2^ showed an increasing pattern after 6 years, with a 6Y–10Y Change of 2.7 (%/year), and rose to its highest value (0.0645) at the end of the follow-up period (Fig 4K), suggesting that men with VFA <100 cm^2^ and V/S ratio ≥0.5 might have a poor renal prognosis with longer follow-up.

### Renal prognosis in patients with VFA < 100 cm^2^ and a V/S ratio ≥0.75 in men and a V/S ratio ≥0.5 in women

**Fig 5:**
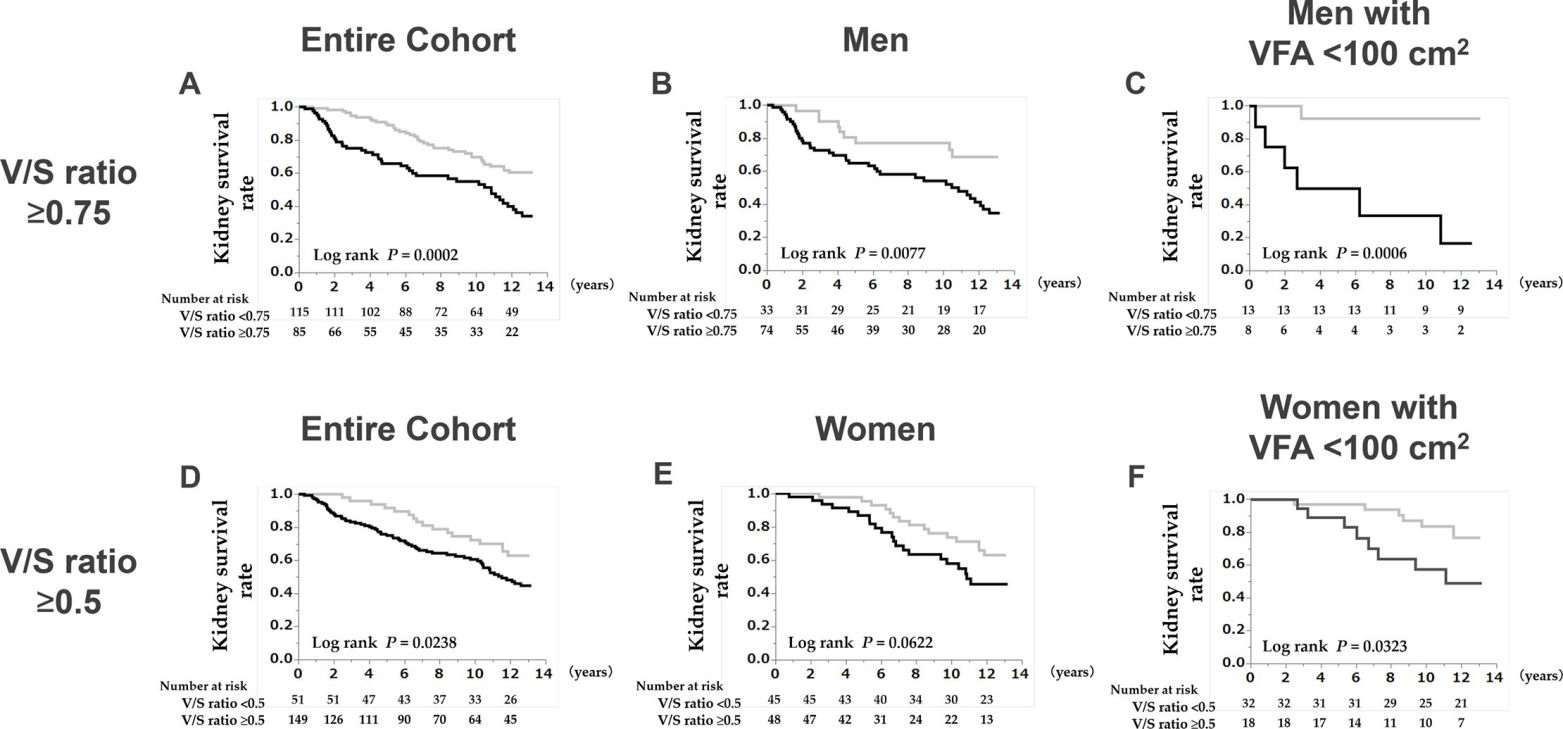
Kaplan–Meier survival curves of the renal prognosis stratified by a V/S ratio ≥0.75, and a V/S ratio ≥0.5 for five cohorts: the entire cohort (A, D), men (B), men with VFA <100 cm^2^ (C), women (E), and women with VFA <100 cm^2^ (F). The renal prognosis for patients meeting any of the V/S ratio criteria is poor. Abbreviations: V/S ratio, visceral to subcutaneous fat ratio; VFA, visceral fat area; *P*, calculated probability.

We evaluated the renal prognosis according to the select cut-off points based on the results for the 6Y–10Y Mean of the pseudo-R^2^ values for the V/S ratio (Fig 5). On Kaplan–Meier analysis, the kidney survival rate was significantly lower in patients with a V/S ratio ≥0.75 in the entire cohort, in men, and in men with VFA <100 cm^2^ (Fig 5C: 10-year kidney survival; V/S ratio ≥0.75 vs. V/S ratio <0.75, 33.3% vs. 92.3%). Similarly, the kidney survival rate was significantly lower in patients with a V/S ratio ≥0.5 in the entire cohort, in women, and in women with VFA <100 cm^2^ (Fig 5C: 10-year kidney survival; V/S ratio ≥0.5 vs. V/S ratio <0.5, 57.4% vs. 83.5%). Compared with that for the larger cohorts (Fig 5A, 5B, 5D and 5E), differences in survival curves for the V/S ratio became larger in the sub-cohorts of patients with VFA <100 cm^2^ (Fig 5C and 5F).

## Discussion

Recently, patient-centered medicine, i.e., treating patients individually according to their heterogeneous characteristics, has attracted increased attention [35, 36]. Thus, the disaggregation of data and analyses of differences within sub-cohorts are required [37, 38]. Furthermore, considering that multifaceted and comprehensive judgments based on abundant medical information are required in the treatment of patients in real clinical settings, there is merit in evaluating various cut-off values in various sub-cohorts, at various follow-up periods, for prognostic factors of importance. In fact, the adequate cut-off value for a risk factor depends on the handling of the risk factor [38–40], the cohort evaluated [38, 39, 41, 42], and the observation period [19, 21].

Fat distribution generally differs between the sexes; men have relatively more visceral fat and women have relatively more subcutaneous fat. Accordingly, the cut-off value for the V/S ratio in terms of the renal prognosis must be evaluated from various perspectives, such as sex differences, differences due to the amount of visceral fat accumulation, and observation-period differences. However, currently, there is no universal agreement on the cut-off point for defining a morbid V/S ratio, and none of the common guidelines is sex specific. Thus, the present study elucidated the significance of the V/S ratio in kidney disease progression in patients with CKD, with consideration of the influence of specific attributes such as sex and the amount of visceral fat accumulation. As the V/S ratio is influenced by sex and fat accumulation, we evaluated interactions between the V/S ratio and sex and VFA category. Furthermore, using the time-series changes in pseudo-*R*^2^ values, we examined not only the efficacy of eight different V/S ratio cut-off points in the entire cohort according to sex but also the efficacy of same eight V/S ratio cut-off points in the sub-cohort of patients with VFA <100 cm^2^ according to sex.

The major findings of the present study are as follows. First, the V/S ratio as evaluated using CT was, for the first time, proved to be associated with CKD progression. Second, VFA category (≥100 cm^2^, <100 cm^2^) significantly interacted with the V/S ratio in terms of the renal prognosis; there was a strong association between the V/S ratio and renal prognosis in patients with VFA <100 cm^2^, but not in patients with VFA ≥100 cm^2^. Interestingly, in patients with VFA <100 cm^2^, the V/S ratio was a more useful renal predictive factor than the VFA itself, when the renal outcome was defined as a ≥30% eGFR decline or the development of ESRD. Third, although sex did not significantly interact with the V/S ratio in terms of the renal prognosis, when stratified by sex, there was a strong association between the V/S ratio and renal prognosis in women but not in men. Furthermore, men and women differed in the critical cut-off points for the V/S ratio in terms of the renal outcome (V/S ratio, 0.75 vs. 0.5). Fourth, on Kaplan–Meier analysis, even in men, the kidney survival rate of patients with a V/S ratio ≥0.75 and VFA <100 cm^2^, was significantly lower than that for patients with a V/S ratio <0.75 and VFA <100 cm^2^. Furthermore, we found that the 6Y-10Y Change for a V/S ratio ≥0.5 in terms of kidney disease progression showed an increasing pattern in men.

From the literature search described in the Introduction section, we identified two articles that evaluated the association between the V/S ratio and CKD progression. In the study by Miura et al., the V/S ratio tended to be associated with 3-year major adverse cardiovascular and cerebrovascular events in 111 patients with acute aortic dissection; however, the V/S ratio was not related to 3-year worsening renal function [43]. Wang et al. reported that a higher V/S ratio was associated with a greater risk for progression to ESRD; however, the V/S ratio failed to reach significance as an independent predictor of diabetic kidney disease progression to ESRD in their cohort of 35 patients with diabetic kidney disease who were followed up for at least 1 year [44]. Although it is unknown why no report has shown a definitive association between the V/S ratio and renal prognosis in patients with CKD, not only the observation period of the study but also the absolute amount of visceral fat accumulation and sex composition might have influenced the results regarding the usefulness of the V/S ratio in terms of the renal prognosis.

In the present study, the V/S ratio was greater in men than in women (1.08 vs. 0.54) and in patients with VFA ≥100 cm^2^ than in patients with VFA <100 cm^2^ (0.97 vs. 0.58), consistent with the results of previous reports [5, 8]. As the V/S ratio is higher in patients with VFA ≥100 cm^2^, and this state is more likely in men, the critical cut-off value of the V/S ratio for renal prognosis in men and in patients with VFA ≥100 cm^2^ may be masked. In the present study, 66.7% of patients with VFA ≥100 cm^2^ had a V/S ratio ≥0.7; thus, there is a possibility that VFA ≥100 cm^2^ itself is a risk factor for a poor renal prognosis, and, as a result, sensitive changes in the V/S ratio may lose their meaning. Consistent with this, when stratified by sex, there was a strong association between the V/S ratio and the renal prognosis in women but not in men, whose VFA, V/S ratio, % of V/S ratio ≥0.7 were all greater than those in women (150.1 cm^2^ vs. 100.1 cm^2^, 1.08 vs. 0.54, 77.6% vs. 21.5%, respectively). Although there were no comments regarding the influence of the absolute amount of visceral fat accumulation, the Framingham Heart Study [5] similarly reported that associations of the V/S ratio with cardiovascular risk factors were stronger in women than in men; additionally, the visceral adipose tissue and V/S ratio were suitably greater in men than in women (2226 cm^3^ vs. 1350 cm^3^, 0.84 vs. 0.39, respectively). However, we consider the low significance of the V/S ratio in men to be superficial and influenced by high visceral fat accumulation. In fact, in men with VFA <100 cm^2^, the kidney survival rate was significantly lower in patients with a V/S ratio ≥0.75 than in patients with a V/S ratio <0.75 (10-year survival; V/S ratio <0.75 vs. V/S ratio ≥0.75, 33.3% vs. 92.3%).

The prognostic ability of potential kidney prognostic factors can depend on the length of the follow-up observation period. Therefore, detailed treatments with consideration of both the short-term and long-term prognoses are desired in real clinical practice. However, until recently, time-series changes in the prognostic ability of prognostic factors have not received much attention. We recently decided to evaluate the time-series changes in pseudo-*R*^2^ values in our clinical studies, based on the fact that the prognostic abilities of risk factors vary with time [19, 21]. The most significant contribution of the pseudo-*R*^2^ values for the renal outcome is the ability to distinguish between short-term and long-term prognostic power. As we reported in a previous study on IgA nephropathy [19], short-term and long-term renal prognostic factors differ. Variables that maintain a high pseudo-*R*^2^ value during follow-up or show an increasing pattern in pseudo-*R*^2^ values over time are considered useful as medium-term and long-term prognostic factors. Long-term renal prognoses tend to be influenced by lifestyle-related diseases, and generally result in time-series increases in the pseudo-*R* ^2^ when patients are not treated successfully. The present study is the first to show a difference in the cut-off value for a renal prognostic factor in accordance with sex or the length of the follow-up observation period, using a time series evaluation of the pseudo-*R*^2^ values. As the medium-term renal prognosis of patients with CKD has not been previously evaluated by pseudo-*R*^2^ values, we attempted to establish indicators for the 6th to 10th years renal prognosis as fixed medium-term indexes that are not influenced by differing follow-up periods in future studies, in order to facilitate comparisons in results between future studies and the present study.

In the present study, although the multivariate Cox regression analyses failed to show an association between the V/S ratio and the renal prognosis in men, the analysis of the pseudo-*R*^2^ values for the renal prognosis showed clear results for men. Furthermore, an interesting pattern in the time-series changes in the pseudo-*R*^2^ values was more clearly observed in men than in women (Table 5B and 5C, values shown in bold): the highest pseudo-*R*^2^ value among the various definitions of a high V/S ratio shifted toward a lower cut-off as the observation period was extended. This result indicates that higher cut-off values are useful for short-term prognosis, whereas lower cut-off values are useful for the long-term prognosis, which is reasonable. Furthermore, among the various definitions of a high V/S ratio, the highest 6Y–10Y Mean of the pseudo-*R*^2^ values was obtained with a V/S ratio ≥0.75 for the entire cohort and the sub-cohort of men, and with a V/S ratio ≥0.5 for the sub-cohort of women. These results may imply a sex difference in the cut-off point for the medium-term renal prognosis. In men, the pseudo-*R*^2^ values for V/S ratios ≥0.7 and ≥0.75 showed an increasing pattern after 6 years, suggesting that men with a V/S ratio ≥0.7 might have a poor renal prognosis with longer follow-up (more than 10 years). Sex-specific cut-off values are required when there are differences in the average value and distribution of the V/S ratio between men and women. On the other hand, the highest value of the 6Y–10Y Mean of the pseudo-*R*^2^ values was obtained with a V/S ratio ≥0.5 for the entire sub-cohort of patients with VFA <100 cm^2^ and for women with VFA <100 cm^2^, and with a V/S ratio ≥0.75 for men with VFA <100 cm^2^. Interestingly, the pseudo-*R*^2^ values for a V/S ratio ≥0.5 in men with VFA <100 cm^2^ showed an increasing pattern after 6 years. Therefore, men with VFA <100 cm^2^ and a V/S ratio ≥0.5 might have a poor renal prognosis with a much longer follow-up period (more than 10 or 20 years).

It has been reported that the clustered number of MetS components is greater than 1.0 in Japanese persons with VFA ≥100 cm^2^ [2], and a VFA ≥100 cm^2^ is a diagnostic criterion for MetS in Japan [1]. In kidney disease, the reported incidence of CKD increases as the number of metabolic components increases [45], and the presence of MetS is associated with ESRD [46]. In the present study, the V/S ratio was a significant renal prognostic factor in the entire cohort and in the sub-cohorts of patients with VFA <100 cm^2^, but not in the sub-cohorts of patients with VFA ≥100 cm^2^. Furthermore, VFA ≥100 cm^2^ significantly interacted with the V/S ratio in terms of the renal prognosis. Considering that visceral fat accumulation itself is an important risk factor in MetS, in a sense, it is not surprising that the VS ratio becomes less important as visceral fat accumulation increases. Studies have reported that patients with VFA ≥100 cm^2^ are at risk for coronary artery diseases [47], cardiovascular diseases [48, 49], and cerebral small vessel diseases [50]. The contribution of each metabolic risk component to renal outcomes may be relatively reduced by the simultaneous presence of interrelated metabolic risk components. On the other hand, even though patients with CKD and VFA <100 cm^2^ are generally considered as low-risk patients (unlike patients with VFA ≥100 cm^2^), little is known about whether patients with VFA <100 cm^2^ have renal risk factors associated with metabolic disorder. The results of the present study are meaningful, in that the V/S ratio was shown to be a more important factor for kidney disease progression in patients with VFA <100 cm^2^ than in patients with VFA ≥100 cm^2^, suggesting that visceral fat-related renal damage has begun in both sexes, even before apparent metabolic-related systemic complications have emerged. Considering the disease characteristics of CKD, which progress over decades, from a public health perspective, prevention and early intervention based on the V/S ratio will likely reduce the burden of kidney disease.

The present study has several limitations. First, the impact of subsequent V/S ratio changes on outcomes was difficult to demonstrate because only baseline laboratory data were used in the analyses. Second, since all the participants were Japanese patients with CKD, the association between the V/S ratio and kidney outcomes may not be generalizable to other populations. Third, a potential selection bias was unavoidable because the patients voluntarily enrolled in this study. Fourth, the men in the present study had lower kidney function and a lower proportion of patients with VFA <100 cm^2^ than did the women. This may have influenced renal prognosis analyses. Fifth, as it was not possible to collect data on the duration of CKD in the present study, it cannot be ruled out that the duration of CKD may have affected the renal prognosis outcome. Additionally, the sample size was relatively small, and further prospective studies with larger cohorts are required to confirm the impact of the V/S ratio on kidney function decline in patients with CKD. On the other hand, the present study has several strengths. First, this is the first study to evaluate the time-series changes in pseudo-*R*^2^ values for the V/S ratio at several cut-off points. Second, the detailed analyses were designed to disaggregate the data stratified by sex and VFA category, which is important for achieving patient-centered medicine [35, 36]. Third, the present study utilized a well-characterized population of Japanese patients with CKD who were treated by nephrologists at a single center using standard CKD care guidelines.

## Conclusions

It may be clinically meaningful to consider the influence of sex and an absolute amount of VFA ≥100 cm^2^ on the V/S ratio in relation to the renal outcome. The present study found, for the first time, that the V/S ratio is associated with CKD progression, especially in patients with VFA <100 cm^2^. Furthermore, based on the 6Y–10Y Mean of the pseudo-*R*^2^ values, the critical cut-off points of the V/S ratio for the medium-term renal prognosis differed between men and women (V/S ratio: 0.75 in men vs. 0.5 in women).
